# Assessment of Oculomotor Functions as a Biomarker in Mild Traumatic Brain Injury

**DOI:** 10.1089/neur.2024.0018

**Published:** 2024-07-03

**Authors:** Ekaterina Lunkova, Jen-Kai Chen, Rajeet Singh Saluja, Alain Ptito

**Affiliations:** ^1^ Department of Neurology & Neurosurgery, McGill University, Montreal, Canada.; ^2^ McGill University Health Centre Research Institute, Montreal, Canada.; ^3^ Montreal Neurological Institute, Montreal, Canada.

**Keywords:** concussion, eye-tracking, fMRI, mild traumatic brain injury, oculomotor functions

## Abstract

Mild traumatic brain injury (mTBI), or concussion, is a major public health problem, and ambiguity still exists regarding its diagnosis. While functional magnetic resonance imaging (fMRI) has been identified as a helpful screening tool for concussion, its limited accessibility in clinical or field settings necessitates a more efficient alternative. Oculomotor function deficit is an often-reported pathology in mTBI. Due to the neuroanatomical overlap between eye-movement circuitry and mTBI pathophysiology, visual deficits are expected. In this study, we investigate the possibility of using an oculomotor assessment tool for finding biomarkers in concussion. We used fMRI with tasks evaluating oculomotor functions: smooth pursuit (SP), saccades, anti-saccades, and optokinetic nystagmus (OKN). Before the scanning, the testing with a system of virtual reality goggles with integrated eye- and head-tracking was used where subjects performed the same tasks as those used in fMRI. Twenty-nine concussed symptomatic adults (CSA) within 1-month postconcussion and 29 age- and sex-matched healthy controls (HCS) were tested to examine blood oxygen level-dependent (BOLD) fMRI alterations associated with performances in oculomotor function after mTBI and evaluate the efficacy of the oculomotor assessment in detecting oculomotor and gaze deficits following mTBI. Comparing CSA with HCS, significant differences were observed in anti-saccades and OKN performance. CSA group exhibited elevated %BOLD signal change on each task compared with HCS: in the superior frontal gyrus during the smooth pursuit, inferior frontal gyrus during the saccades, putamen and dorsolateral prefrontal cortex (DLPFC) during the anti-saccades, and lingual gyrus and IFG during the OKN. Key findings include the following: (1) oculomotor deficits in concussed subjects compared with controls, (2) abnormal activation patterns in areas related to the regulation and control of oculomotor movements, suggesting concussion-induced disruptions, and (3) the potential of oculomotor assessment as a promising approach for mTBI biomarkers, with anti-saccades and OKN identified as the most sensitive tasks.

## Introduction

Mild traumatic brain injury (mTBI), often used synonymously with concussion, is a major public health problem. mTBI can be a risk factor for, among others, a decline in cognitive functions,^1^ early dementia,^2,3^ and mental illness,^4^ creating serious challenges for the society and economy, especially in terms of its impact on health care costs and workforce productivity. Ambiguity still exists regarding the pathophysiology and management of concussions, and hence, establishing an objective diagnosis remains a topical problem. Previous studies by our team demonstrated that functional magnetic resonance imaging (fMRI) is an objective approach that allows consistent, reproducible results as a screening tool for concussion.^5,6^ However, given the limited availability of fMRI in clinical or on-the-field settings, a more efficient and less resource-consuming approach is needed.

Oculomotor dysfunction is a prevalent condition in patients who have suffered an mTBI, with up to 90% exhibiting impairments in the brain’s ability to coordinate eye movements with accuracy and control.^7,8^ Postconcussion, individuals frequently report experiencing oculomotor symptoms, such as blurred vision, convergence insufficiency, diplopia, difficulty reading, headaches, dizziness, nausea, general asthenopia, and impaired ability to scan visual information. In addition, they may experience difficulties in tracking moving objects.^9,10^ The oculomotor symptoms experienced by individuals postconcussion may lead to functional impairments, such as difficulties reading, decreased academic performance, and cognitive impairments.^9,10^ These functional impairments can be attributed to the disruption of the underlying neurophysiology of oculomotor functions caused by concussion.^11^

mTBI may be a leading cause of clinically impaired smooth pursuit and saccadic eye movements.^9^ Performance in an anti-saccades task was shown to be impaired in concussed subjects; these subjects also showed hyperactivation in the cerebellum, primary and secondary visual cortex, and visual area V5/MT.^12^ Furthermore, previous studies with subjects who sustained an mTBI compared with controls have demonstrated increased activation in these areas while producing saccadic movements. A study by Hecimovich et al. with college rugby players tested before and after the season showed significant differences between concussed and nonconcussed groups for total saccades, with differences from baseline to follow-up observed for saccade velocity in both groups.^13^ Even 3–6 months after mTBI, patients with prolonged postconcussive symptoms demonstrated impaired production of anti-saccades, memory-guided saccades, and self-paced saccades, in comparison with fully recovered patients.^14–16^

Astafiev et al. demonstrated that smooth pursuit may exhibit increased variability and be susceptible to disruption under higher cognitive loads, which corresponds to differences in activation of the right inferior frontal gyrus (IFG) and basal ganglia.^17^ Another study by Johnson and colleagues showed that during smooth pursuit eye movements postconcussion, increased activation was observed in the cerebellum, frontal lobes, and visual cortices, although no significant differences were observed in the performance of the concussed group compared with healthy individuals.^12^ Increased activation may be explained by compensatory mechanisms in which functional changes in brain resources contribute to correct task performance without permanent alterations in networks.^12^ Overall, the findings on the smooth pursuit eye movements postconcussion remain ambiguous.

One of the other eye movements commonly evaluated by eye-tracking systems is optokinetic nystagmus (OKN). As OKN consists of two components—(1) smooth pursuit and (2) saccades, it can be suggested that similar patterns of brain activation observed in individuals with mTBI during smooth pursuit and saccadic eye movements may also be present during OKN.^18^ A study by Wright and colleagues (2017) showed that the integrity of visual processing related to optokinetic stimulation is compromised in a manner that consistently induces symptoms among individuals experiencing concussion.^19^ In addition, individuals with mTBI exhibit heightened symptomatology, encompassing dizziness, headache, and nausea, following optokinetic reflex testing in contrast to healthy subjects.^19,20^ Hence, optokinetic reflex testing emerges as a potentially valuable diagnostic modality for mTBI.

Due to the high speed of saccadic and pursuit movements, oculomotor impairments are often missed during clinical examinations, highlighting the need for sensitive screening tools in mTBI diagnosis, such as eye-tracking systems.^21^ Recent findings demonstrated that eye-tracking metrics correlate with concussion symptoms and can detect convergence and accommodative abnormalities associated with concussion. Therefore, the use of an eye-tracking system as a rapid, objective, and noninvasive tool for diagnosing mTBI appears to be warranted.^22^ Furthermore, considering the highly variable recovery trajectory following mTBI, it is crucial to utilize reliable and objective oculomotor function tests to monitor patient outcomes. Noninvasive eye-tracking experiments using these techniques are widely used across various research domains, such as vision science, psychology, sport and exercise sciences, automotive sciences, marketing, and the gaming industry.^8,23^

Of note, not all concussed subjects complain of eye-movement-related symptoms, which suggests that some of them have preserved oculomotor functions. In everyday life, this deficit can go unnoticed; however, under increased workload condition (i.e., in sports) these subjects’ difficulties may surge. Thus, it is of utmost importance to use more sensitive oculomotor tasks that have the potential to reveal more profound deficits. Data collection from a comprehensive combination of sensory and motor circuits will shed light on the underlying mechanisms of oculomotor function and the extent of their vulnerability to mTBI.

In this study, we aimed to do the following:

1. Examine blood oxygen level-dependent (BOLD) fMRI alterations associated with performances in oculomotor function after mTBI (evaluating saccades, anti-saccades, smooth pursuit, and OKN).
2. Evaluate the efficacy of the oculomotor assessment in detecting oculomotor and gaze deficits following mTBI.

## Materials and Methods

### Participants

Twenty-nine concussed symptomatic adults (CSA) within one month (mean number of days postinjury = 28.2, SD = 9.9) postinjury were selected according to the WHO task force criteria [21 (72.4%) females, mean age = 28.3, SD = 10] and a group of 29 sex- and age-matched adult healthy control subjects (HCS) (mean age = 29.1, SD = 9.7) without a history of neurodevelopmental or neurological disorders, head injuries, attention deficit and hyperactivity disorder (ADHD), and/or presence of significant abnormalities seen on structural magnetic resonance imaging (MRI) scans (assessed by a clinician) were included in the study. Mechanism of injury in the CSA group included motor-vehicle accidents (MVA), skiing/snowboarding, and general (e.g., slip and fall). Concussed subjects were identified as symptomatic/asymptomatic according to their results on the Postconcussion Symptom Scale (PCSS), and only symptomatic subjects were included in the study (PCSS score > 21/132; mean score = 44 ± 24).

#### Oculomotor functions assessment using the VR-goggles eye-tracking system

The oculomotor evaluation was conducted before MRI scanning using virtual reality (VR) goggles equipped with binocular recordings in 3D (horizontal, vertical, and pupil size) and head recordings in 6D (3D angular and 3D linear accelerations). These were recorded concurrently for eye and head angles at a 100 Hz sampling rate. The visual displays within the goggles were generated via a laptop, followed by ocular and head data recordings by the goggles. Eye and head movements were evaluated in response to visual and vestibular stimuli, or lack thereof (e.g., to evaluate spontaneous nystagmus), to detect deviations from normal eye and head responses of healthy subjects. The full evaluation consisted of a battery of tests that takes less than 10 min to administer, including three head-free conditions (smooth pursuit [head-free], active visual vestibulo-ocular reflex [VOR, horizontal], and active visual VOR [vertical]), and five head-fixed conditions (smooth pursuit [head-fixed], saccades, anti-saccades, OKN, and spontaneous nystagmus) (Table 1). Afterward, four tasks—smooth pursuit, saccades, anti-saccades, and OKN—were repeated during fMRI to measure brain activation associated with performances on the tasks. Only head-fixed conditions were chosen for use inside the MRI scanner.

**Table 1.** Eye–Head Coordination Tests and Measured Variables with Units

	System of interest (protocol)	Measured aspect of metrics
1	Saccades (flashed targets, self-paced)	Delay (ms) Accuracy (degrees)
		Generation rate (sac/sec) Main sequence (peak velocity vs. duration)
2	Anti-saccades	Accuracy (degrees)
		Latency (ms)
3	Active head-fixed or head-free passive VOR active VOR, pursuit, OKN	Mean vergence over the whole protocol period (sac/min)
		Vergence for each phase of movement (saccade and fixation; degrees)
4	Nystagmus during active gaze shifts head-fixed or head-free Spontaneous nystagmus in the dark Vestibulo-ocular reflex Optokinetic nystagmus	Asymmetry of peak response, phase lag (%)
		Full response characterization in both phases with numeric parameters
		Generation frequency
		Tracking error, gaze stabilization (degrees)
5	Head-free gaze shifts	Eye vs. head contributions
6	2D Target tracking head-fixed or head-free smooth pursuit and corrective saccades	Accuracy in different initial positions (degrees)
		Corrective saccade rate (sac/sec)
		Response symmetry
7	Pupil size	Diameter (mm)

ms, millisecond; sac/sec, saccades per second; sac/min, saccades per minute; VOR, vestibulo-ocular reflex; OKN, optokinetic nystagmus.

### Image acquisition

All scanning was performed on a Siemens 3 Tesla MRI system equipped with a 64-channel head coil at the Montreal Neurological Institute (MNI) BIC MRI platform. First, T_1_-weighted images were acquired for anatomical reference (3D MP-RAGE, TR = 2300 ms, TE = 2.98 ms, 176 slices, slice thickness = 1 mm, FOV = 256 mm, image matrix = 256 × 256, flip angle = 9 degrees, interleaved excitation) for fMRI data. fMRI data were acquired using BOLD activation studies with T2*-weighted GE-EPI (TR = 3000 ms, TE = 30 ms, 38 slices, slice thickness = 4 mm, FOV = 256 mm, image matrix = 128 × 128, interleaved excitation).

### Oculomotor tasks used in fMRI

We used task-based fMRI with 4 tasks evaluating oculomotor functions: (1) **Smooth Pursuit**: subjects were asked to follow a moving target (dot) with their eyes only; (2) **Saccades:** subjects were told to look at a target (dot) as it jumped around on the screen with their eyes only; (3) **Anti-saccades**: subjects had to look at the dot at the center of the screen—when a red X appeared, they had to avoid looking at the red X and instead orient their eyes into the opposite field of view in the same location—then follow the dot back to the center; (4) **OKN**: subjects were asked to pick a dot and follow it until it left their field of view, and to continue in the same manner with each subsequent dot; and (5) **Baseline condition**: (a) Before each task, the baseline condition was presented to the subjects (for conditions (1), (2), and (3), it was a fixed dot at the center of the screen for a duration of 12 sec; for condition (4), it was a fixed field of dots for a duration of 15 sec). Each of the conditions lasted 30 sec, while subjects were head-fixed and asked to complete the tasks by moving their eyes only. Two identical functional scanning sessions were conducted sequentially. Each scanning session lasted 6 min and consisted of two runs of the set of four tasks. The subjects underwent extensive training before the scanning to ensure familiarity with the tasks. These tasks were selected because they require the head to be fixed throughout the protocol duration, in the same manner as during MRI scanning. Other head-free conditions in the oculomotor screening battery were therefore left out (head-free smooth pursuit, VOR vertical and horizontal). One out of five head-fixed tasks was not used during fMRI—spontaneous nystagmus—because it was recently added to the oculomotor screening battery and lacks normative data.

### Behavioral analysis (oculomotor and gaze assessment)

The data from the VR goggles and the eye-tracking system were automatically gathered and processed by the NeuroFlex^®^ software system. The results for each subject included all metrics enumerated in Table 1, and the deviations of the results (if any) were described in individual reports. Mean values, standard deviations (SDs), and normative range of the results in the group of HCS were calculated. The normative range was counted as mean ± 2SD.

### Neuropsychological assessment

Neuropsychological assessment was used to identify the correlation between the severity of cognitive functional impairment, the imaging findings, and performance on the oculomotor tasks. The domains included in the neuropsychological assessment were selected according to their sensitivity to mTBI as demonstrated in previous studies: attention, working memory, processing speed, and problem-solving.^24^ The neuropsychological assessments consisted of the following tests: Verbal Working Memory Task (M. Petrides), Rey Auditory Verbal Learning Test (RAVLT), Trial Making Test (TMT), Purdue Pegboard, Tower of London, WAIS-IV Processing Speed Index Subtests (symbol search, coding), Symbol Digit Modalities Test (SDMT). The neuropsychological tests are conducted to determine if cognitive difficulties are related to structural and hemodynamic alterations identified by MRI sequences and/or to oculomotor problems.

### Questionnaires

CSA were asked to fill out the following questionnaires before participating in the study: PCSS, Beck Anxiety Inventory (BAI), Beck Depression Inventory II (BDI-II), and Dizziness Handicap Inventory (DHI). PCSS was used to identify symptom severity, and BAI and BDI-II scores were used to identify the possible presence of depression/anxiety and its effect on task performance.

### Data processing and statistical analysis

#### MRI processing

Task-based fMRI. All MRI images were preprocessed and analyzed using SPM12.^25^ During the preprocessing stage, all the functional images were realigned and unwrapped; slice-time corrected; coregistered to a T1-weighted reference image; structurally and functionally normalized and segmented into gray matter, white matter, and cerebral spinal fluid (CSF) tissue; and smoothed using a 6 mm Gaussian kernel. The preprocessed images for each subject then underwent first-level analysis, where the model was specified and estimated for the two runs of the tasks. The model was specified using the conditions and onset times for each task. To determine an alteration of the level of BOLD signal specific to each task, the contrasts were identified between the task condition and the baseline condition. Afterward, second-level analysis was conducted to identify BOLD signal changes during each task, respectively, for the HCS and CSA groups. Exploratory brain analysis resulted in whole-brain activation maps. Between-group comparison of whole-brain maps was conducted using a 2-sample *t*-test with family-wise error (FWEr) correction for *p* values. Afterward, regions of interest (ROIs) were extracted from whole-brain analysis; before this, the sphere of 5 mm radius was used to create a mask for each ROI using the MarsBaR toolbox (ROIs were based on the results of 31 healthy controls [HCs] and previous findings using similar tasks).^26,27^ Afterward, a comparison of the BOLD signal in each ROI between two groups was performed (data checked on normality—data are normally distributed, hence using a 2-sample *t*-test). Using the results of HCS extracted from ROIs, 95% confidence intervals were determined to establish a “normal range” % of BOLD signal change.

#### Oculomotor assessment

Oculomotor data were gathered using the VR-goggles eye-tracking system and was automatically processed through the NeuroFlex^®^ software. The results for each subject included all the metrics demonstrated in Table 1, and deviations of the results (if any) were identified in individual reports. Data for each metric were compared using 2-sample *t*-test and U-test, depending on distribution.

#### Correlation between oculomotor metrics and BOLD signal alterations

A multiple regression analysis was used to determine a relationship (if any) between the level of BOLD signal in each ROI and each of the oculomotor metrics of the four tasks used in fMRI. %BOLD signal change in each ROI was entered as a dependent variable, and oculomotor metrics were entered as independent variables; all values were converted to z-scores before the implementation of multiple regression.

#### Neuropsychological assessment and questionnaires

Neuropsychological assessment results were converted to standard scores, and several subjects with scores below the normal range were identified according to the norms for each task^23^; the conclusions were therefore qualitative in nature. The results on the questionnaires were assessed according to the individual guidelines for each.

#### Correlation between neuropsychological assessment and questionnaire results and imaging findings

To determine correlations with PCSS, BAI, BDI-II, and DHI scores, as well as scores on the cognitive tests, multiple regression analyses were implemented. %BOLD signal change in each ROI was entered as a dependent variable, and results on neuropsychological tests and questionnaires were used as independent variables. All values were converted to z-scores before the implementation of multiple regressions.

Oculomotor and BOLD signal analyses and descriptive statistics as well as additional statistical analyses were performed using the IBM Statistical Package for the Social Sciences (SPSS) version 28.0.1.1 for Mac OS.

## Results

### Oculomotor tasks

**FIG. 1.**
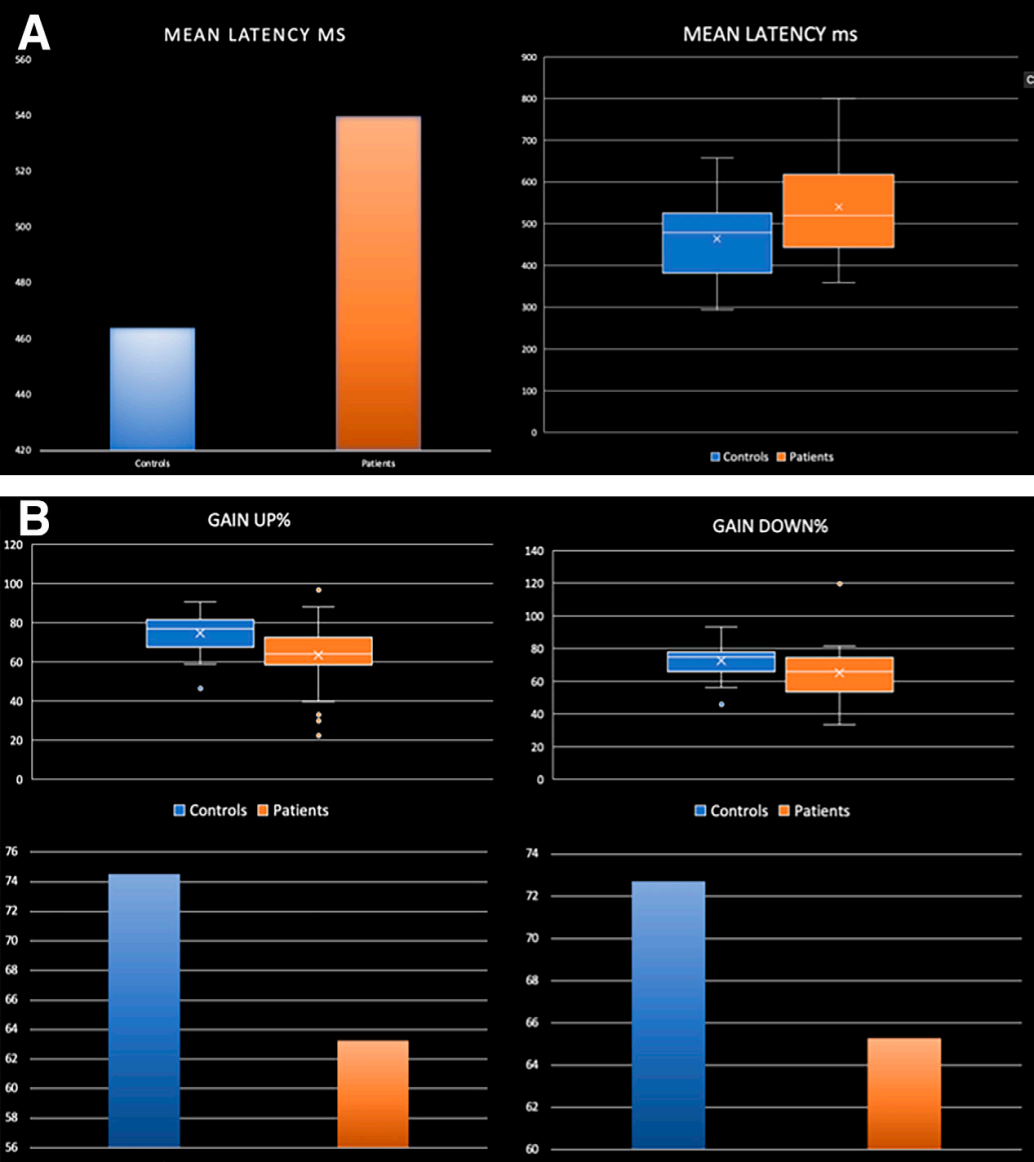
**(A)** Differences between concussed subjects and healthy controls on the mean latency metric of the anti-saccades task. **(B)** Differences between concussed subjects and healthy controls on the Gain Up/Gain Down metrics of the optokinetic nystagmus task.

Compared with HCS, CSA showed significant differences in performance on two oculomotor tasks (anti-saccades and OKN: Fig. 1). During the anti-saccade task, they showed significantly higher mean latency between time of stimuli presentation and time of eyes onto target (*p* = 0.012, d = 0.68). During the OKN task, the mean eye velocity relative to target velocity was significantly lower than HCS in the up (*p* = 0.003, d = 0.826) and down (*p* = 0.046, d = 0.573) directions (Table 2). The results on all the other metrics did not indicate statistically significant differences (Table 2).

**Table 2.** Number of CSA Outside of the Normal Range

	CSA (*n* = 29)
SP	1 (3.5%)
VOR (horizontal)	9 (31%)
VOR (vertical)	8 (27.6%)
Saccades	9 (31%)
Anti-saccades	11 (37.9%)
OKN	15 (51.7%)

CSA, concussed symptomatic adults; SP, smooth pursuit; VOR, vestibulo-ocular reflex; OKN, optokinetic nystagmus.

### fMRI results

**FIG. 2.**
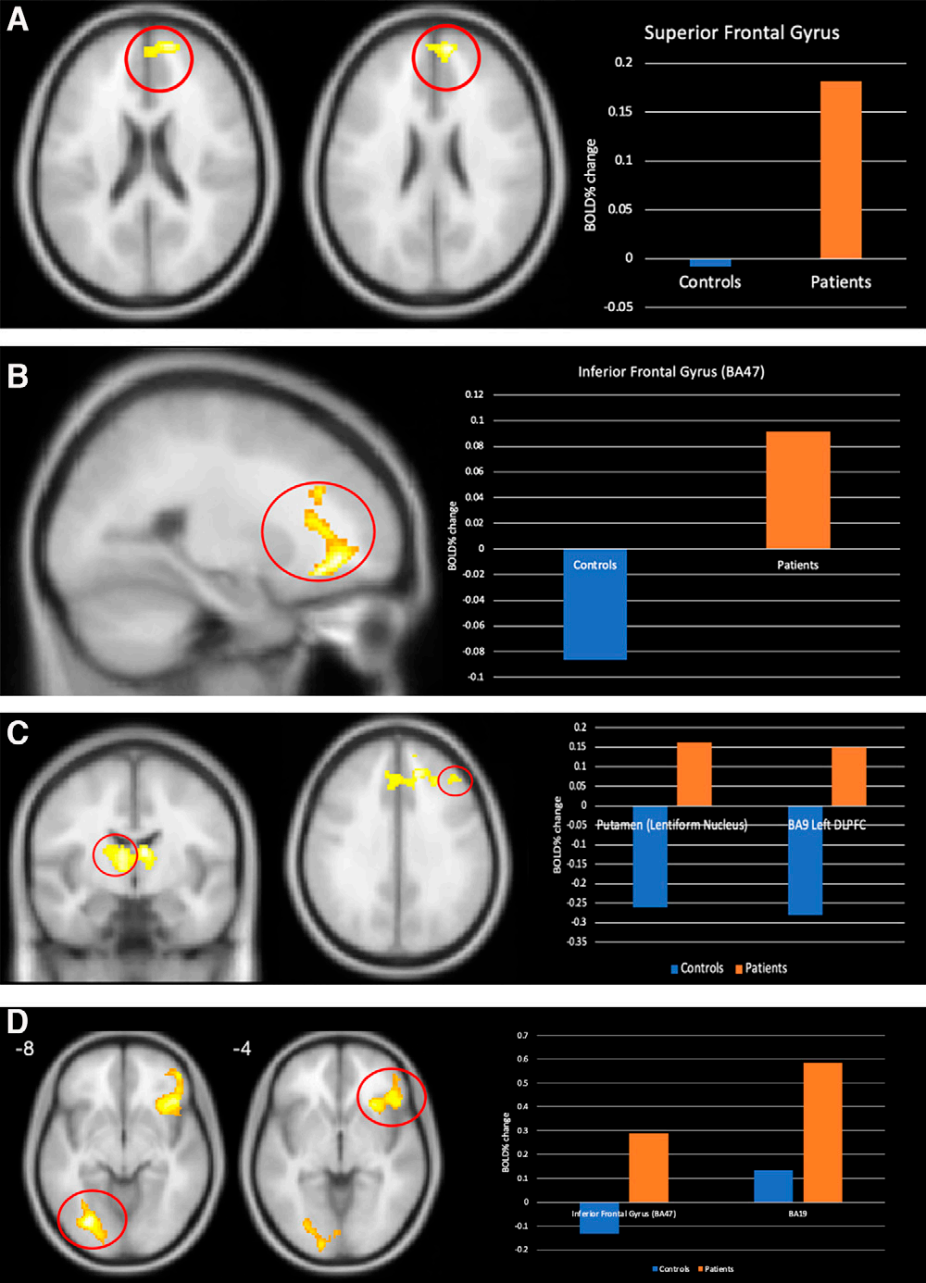
Areas of higher BOLD signal change in concussed subjects compared with healthy controls, with graphs indicating the size of the % BOLD signal change during: **(A)** smooth pursuit, **(B)** saccades, **(C)** anti-saccades, and **(D)** optokinetic nystagmus. BOLD, blood oxygen level-dependent.

According to the results of whole-brain analysis, the CSA group appeared to have elevated %BOLD signal change on all four tasks compared with the HC group. The mTBI group had higher activation in the right superior frontal gyrus (SFG) compared with the HCS group (Fig. 2A, *p* = 0.018, d = 1.012) during the smooth pursuit task, in the left IFG during the saccades task (Fig. 2B, *p* = 0.048, d = 0.841), in the right putamen (Fig. 2C, *p* = 0, d = 1.035) and left DLPFC (Fig. 2C, *p* = 0, d = 0.962) during the anti-saccades test, and in the left lingual gyrus (Fig. 2D, *p* = 0.005, d = 0.937) and right IFG (Fig. 2D, *p* = 0.013, d = 0.945) during the OKN task. ROI analysis, in turn, did not show any significant differences between the groups.

### Neuropsychological assessment

The most difficulties experienced by the CSA group were observed on tasks measuring working memory and learning (44.8% of CSA had results below the normal range on “Learning” part of RAVLT), visual attention and task switching (Trail Making Test; 48.3% of CSA had results below the normal range), and graphomotor processing speed (SDMT; 44.8% of CSA had results below the normal range; Table 3).

**Table 3.** Number and % of CSA with Results Outside of the Normal Range

	CSA with results outside of the normal range (*n* = 29)
RAVLT learning	13 (44.8%)
RAVLT immediate recall	6 (20.7%)
RAVLT delayed recall	7 (24.1%)
RAVLT recognition	7 (24.1%)
Pegboard	6 (20.7%)
Processing speed index	6 (20.7%)
Trail Making Test Part A	8 (27.6%)
Trail Making Test Part B	14 (48.3%)
Tower of London Accuracy	6 (20.7%)
Tower of London Speed	3 (10.3%)
Symbol Digit Modalities Test	13 (44.8%)

CSA, concussed symptomatic adults; RAVLT, Rey Auditory Verbal Learning Test.

### Questionnaires

The majority (79%) of the concussed subjects showed minimal to mild symptoms of depression (BDI-II mean score = 14.1 ± 9.3), and more than a half of the subjects (55%) showed minimal to mild symptoms of anxiety (BAI mean score = 17.3 ± 14.2). In addition, most of the subjects (79%) showed mild to severe symptoms of dizziness and unsteadiness (DHI mean score = 32.6 ± 21.4; Table 4).

**Table 4.** Results of the BAI, BDI-II, and DHI Questionnaires

	Number and % of CSA (*n* = 29)
BDI-II	
Normal	18 (62%)
“Mild depression”	5 (17%)
“Moderate depression”	4 (13.8%)
“Severe depression”	2 (6.9%)
BAI	
Normal	9 (31%)
“Mild anxiety”	7 (24.1%)
“Moderate anxiety”	7 (24.1%)
“Severe anxiety”	6 (20.7%)
DHI	
Normal	6 (20.7%)
“Mild handicap”	12 (41.4%)
“Moderate handicap”	5 (17%)
“Severe handicap”	5 (17%)

CSA, concussed symptomatic adults; BAI, Beck Anxiety Inventory; BDI-II, Beck Depression Inventory II; DHI, Dizziness and Handicap Inventory.

### Correlation between the neuroimaging findings, performance on neuropsychological assessment and oculomotor tests, and results on the questionnaires

Results of multiple regression analyses showed a strong correlation between one of the ROIs of the anti-saccades task—the precuneus—and the results on the DHI (r^2^ = 0.672, *p* = 0.037). There was also a correlation between performance on the anti-saccades task (in particular, in the percentage of success in eyes orienting in the correct direction) and the processing speed index of concussed subjects in neuropsychological assessment (r^2^ = 0.454, *p* = 0.019). There was no correlation between neuroimaging findings and performance on the oculomotor tasks, neuropsychological assessment results, and other questionnaires.

## Discussion

According to the fMRI findings in our study, CSA demonstrated an increase in % BOLD signal change compared with HCS during all four tasks presented during fMRI: in the right SFG during smooth pursuit, in the left IFG during saccades, in the right putamen and left DLPFC during anti-saccades, and in the left IFG and right lingual gyrus during OKN. Of all the oculomotor tasks, only performances by concussed subjects on the anti-saccades and OKN tasks were significantly different from HCS, with CSA showing significantly lower mean eye velocity when following the target. Overall, the atypical activation patterns observed, taken together with uncharacteristic task performances by the concussed subjects, point to functional disruption in the post-mTBI brain.

### Smooth pursuit and saccades

In CSA, all tasks showed increased activation patterns in frontal areas, which could be associated with either an enhanced cognitive effort invested in task implementation and/or the engagement of compensatory neural resources to support eye movement production postinjury. More important, involvement of broader and additional areas during the oculomotor tasks postconcussion is in keeping with previous findings.^28,29^ During smooth pursuit, an increase in % BOLD signal change in the right SFG, the area that is suggested to be related to the regulation of spatial attention and visuospatial processing,^30^ is observed. During saccades—the increase in the left IFG is thought to monitor errors, control inhibition, and regulate attention.^31^ Concurrently, there was no apparent deficit in task performances during these tasks, suggesting that the increased activation associated with these tasks may be due to utilization of compensatory mechanisms where transient alterations of brain resources ensure proper task performances without permanently altering function.^32^ It has also been proposed that eye movements are so fundamental that they could be resilient to concussion,^28^ hence the “normal” performances on the tasks requiring these eye movements. The study by Zhang also documented a lack of difference in performances postconcussion during smooth pursuit or saccades production, in addition to showing additional activations, especially in frontal areas.^28^

### Anti-saccades and OKN

In contrast, CSA had difficulties during the anti-saccades and OKN tasks, showing not only a higher mean latency between the time of stimulus presentation and time to eyes on target (anti-saccades) and lower mean eye velocity relative to target velocity when the target moves up/down (OKN), but also elevated % BOLD signal changes compared with HCS in broader areas than during the smooth pursuit and saccades tasks. Such a distinction could be related to anti-saccades and OKN being more challenging than smooth pursuit or basic saccadic eye movements.^29^ During anti-saccades, increased activation was found in DLPFC, the area commonly shown to have altered activation patterns in CSA, especially during verbal and visual working memory tasks.^5,6^

The involvement of the DLPFC in decision-making processes is well-established. Anti-saccadic eye movements, which require voluntarily redirecting gaze away from a target, necessitate the engagement of various cognitive processes, including inhibitory and attentional control, cognitive flexibility, and the suppression of reflexive eye movements. These cognitive processes are closely linked to executive functions, underlining the significant contribution of the DLPFC in executing these complex oculomotor tasks. In fact, concussion could impair inhibition and executive control 30 days postinjury, despite the brain’s attempt to implement compensatory strategies for achieving task goals.^28^ A study by Slobounov and colleagues on concussed patients also showed increased activation in DLPFC during spatial encoding, indicating a greater effort compared with HCs.^33^

Increased activation during anti-saccades was also observed in the putamen, a novel finding not discussed in previous studies with concussed subjects tested on oculomotor functions. The putamen plays a role in motor planning and coordination and contributes to selecting and inhibiting specific eye movements, integrating sensory information related to eye position and movement, and adjusting gaze based on cognitive and motor demands.^34^ Increased activity in the DLPFC and putamen could reflect a heightened cognitive effort to support the inhibition of reflexive eye movements and the execution of anti-saccades.

**FIG. 3.**
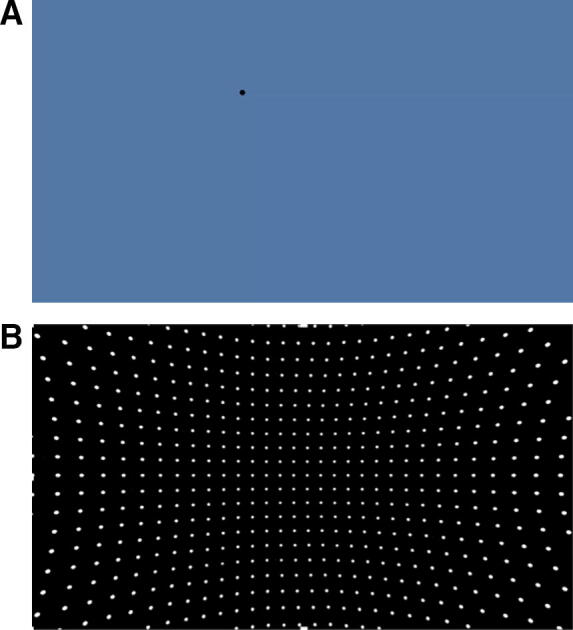
**(A)** The interface during the smooth pursuit, saccades, and anti-saccades task implementation. **(B)** The interface during the OKN task implementation. OKN, optokinetic nystagmus.

Only during the execution of OKN did we see a higher % BOLD signal change in CSA relative to HCS in both the frontal and occipital areas. There was an increase in activation in the right IFG, the area opposite the one activated during saccades production, as well as in the left lingual gyrus. The right IFG is thought to control premature or no longer appropriate motor response inhibition.^35,36^ A possible explanation for the types of patterns we observed could be related to the fact that OKN is the last task of the set we used, as well as the most different from the others [there are multiple moving dots on a black background (Fig. 3A) as opposed to a single dot and blue background (Fig. 3B)], and CSA struggled when adapting new strategies for task implementation. A similar explanation could be applied to the increase in % BOLD signal change in the lingual gyrus, which plays a role in higher level analysis and interpretation of visual stimuli, including motion processing and integration of visual information with other cognitive processes.^37^

The OKN task is known to impose higher visual demands compared with other tasks. It necessitates a more intense suppression of irrelevant stimuli, such as other moving dots, which concussed subjects reported as triggering physical symptoms such as brief vertigo and light-headedness. Consequently, these observations suggest that heightened activity in both the frontal and occipital areas during eye movements in concussed individuals may indicate increased cognitive effort in supporting the coordination and execution of saccadic and smooth pursuit eye movements, which are fundamental components of the OKN task.

### Limitations

This study included subjects who suffered mTBI one to five weeks before their participation in the study, as well as subjects with different approaches to rehabilitation (e.g., resting at home for several weeks or coming back to work a few days after the injury), which could affect variability in performance on oculomotor tasks, with subjects showing a different extent of recovery. In addition, the present study is constrained to individuals surpassing a symptom cutoff score on the PCSS, thus limiting the generalizability of findings to all cases of mTBI.

## Conclusions

Overall, we can highlight three main findings from our study. First, CSA showed oculomotor deficits compared with HCS: their eye velocity was significantly slower relative to target velocity. Second, areas with atypical activation patterns in CSA during task-based fMRI are primarily associated with regulation and top-down control of the oculomotor movements, suggesting that concussion, in fact, disrupts oculomotor functions. Finally, our results suggest that oculomotor assessment is a promising approach for determining mTBI biomarkers, with two tasks most sensitive to concussion—anti-saccades and OKN. Using these two tasks with an eye-tracker as a potential oculomotor biomarker of concussion could have significant implications for the future of concussion diagnosis. Such an approach could be used for establishing return to work, study, or play guidelines and potentially help prevent premature return to activities that could lead to slower recovery and increased vulnerability to repeat injury.

## Ethics statement 

We obtained approval for this study from McGill University Institutional Review Board IRB00010120. Written informed consent was obtained from all participants involved in the study. The consent form outlined the purpose of the research, the procedures involved, and the potential risks and benefits. Participants were informed of their right to withdraw from the study at any time without consequences.

## Data Availability

The data are available on request from the corresponding author.

## Abbreviations Used

ACC: Anterior Cingulate Cortex
BAI: Beck Anxiety Inventory
BDI-II: Beck Depression Inventory
CEF: Cingulate Eye Field
CI: Confidence Interval
CSA: Concussed Symptomatic Adults
CSF: cerebrospinal fluid
DHI: Dizziness Handicap
